# Prognostic Implications of Preoperative N-Terminal Pro-B-Type Natriuretic Peptide Dynamics in Patients Undergoing Cardiac Surgery

**DOI:** 10.1016/j.jacadv.2025.102096

**Published:** 2025-08-26

**Authors:** Leo Pölzl, Ronja Lohmann, Fabian Theurl, Christian Puelacher, Philipp Sterzinger, Jonas Eder, Christian Sutter, Maria Ioannou-Nikolaidou, Felix Nägele, Jakob Hirsch, Michael Graber, Clemens Engler, Vanessa Heim, Sophia Schmidt, Juliane Kilo, Sebastian J. Reinstadler, Hanno Ulmer, Samuel Heuts, Tomas Gudbjartsson, Anders Jeppsson, Emely Ögren, Andrea Griesmacher, Michael Grimm, Axel Bauer, Nikolaos Bonaros, Gerhard Pölzl, Johannes Holfeld, Can Gollmann-Tepeköylü

**Affiliations:** a Department of Cardiac Surgery, Medical University of Innsbruck, Innsbruck, Austria; b University Clinic of Internal Medicine III, Cardiology and Angiology, Medical University of Innsbruck, Innsbruck, Austria; c Department of Statistics, University of Warwick, Coventry, United Kingdom; d Department for Medical Statistics, Informatics and Health Economics, Medical University of Innsbruck, Innsbruck, Austria; e Cardio-Thoracic Surgery Department, Maastricht University Medical Centre, Maastricht, the Netherlands; f Department of Cardiothoracic Surgery, Landspitali University Hospital, Faculty of Medicine, University of Iceland, Reykjavik, Iceland; g Department of Molecular and Clinical Medicine, Institute of Medicine, Sahlgrenska Academy, University of Gothenburg, Gothenburg, Sweden; h Department of Cardiothoracic Surgery, Sahlgrenska University Hospital, Gothenburg, Sweden; i Central Institute of Clinical Chemistry and Laboratory Medicine, Medical University of Innsbruck, Innsbruck, Austria; j Department of Cardiovascular Surgery, German Heart Center Munich, School of Medicine & Health, Technical University of Munich, Munich, Germany

**Keywords:** cardiac surgery, NT-proBNP, perioperative risk assessment

## Abstract

**Background:**

High levels of N-terminal prohormone of brain natriuretic peptide (NT-proBNP) reflect poor cardiac status in heart failure patients.

**Objectives:**

This study analyzed the association of preoperative NT-proBNP dynamics with 30-day and 5-year mortality after cardiac surgery.

**Methods:**

A consecutive cohort of 6,938 patients undergoing cardiac surgery was analyzed. The relationship between preoperative NT-proBNP levels and 30-day and 5-year mortality (median follow up time: 4.53 [2.00-5.00] years) adjusted for EuroSCORE II was explored with a Cox proportional hazards model. The dynamics of preoperative NT-proBNP levels were analyzed by comparing the values at diagnosis or assignment to surgery with the values on the day before surgery (n = 4,739). Results were validated in an external cohort from the SWEDEHEART registry (n = 3,117).

**Results:**

Median preoperative NT-proBNP concentration was 552 (208-1,591) ng/L. Death within 30 days occurred in 2.1% (149/6,938) of the population. High preoperative NT-proBNP levels were associated with higher 30-day and 5-year mortality. Initial high NT-proBNP at diagnosis, with subsequent decrease in preoperative NT-proBNP below 3,000 ng/L, was associated with more favorable perioperative outcomes after adjustment for EuroSCORE II shorter stays in intensive care unit (OR: 0.60, 95% CI: 0.44-0.82), less use of ultrafiltration (OR: 0.48, 95% CI: 0.33-0.70), or extracorporeal membrane oxygenation (OR: 0.26, 95% CI: 0.12-0.57; all *P* < 0.001) and lower 30-day mortality (HR: 0.21, 95% CI: 0.07-0.61; *P* = 0.004). Five-year survival was improved in patients with decreases in preoperative NT-proBNP levels (log-rank: *P* < 0.001, HR: 0.44, 95% CI: 0.30-0.65).

**Conclusions:**

Reductions in NT-proBNP levels before surgery were associated with lower 30-day and 5-year mortality after cardiac surgery. Patients with high NT-proBNP concentrations may benefit from preoperative optimization to lower NT-proBNP.

N-terminal prohormone of brain natriuretic peptide (NT-proBNP) is synthesized as an inactive prohormone, which is then cleaved to release the active hormone BNP and the inactive N-terminal fragment (NT-proBNP) in response to increases in cardiac filling pressures, such as those observed during congestive heart failure or following myocardial ischemia.^1^^,^^2^ High NT-proBNP levels are independently associated with mortality and adverse cardiovascular outcomes in patients undergoing elective noncardiac surgery, with and without cardiac failure.^3^, ^4^, ^5^, ^6^

Numerous studies have reported an association of perioperative NT-proBNP levels with postoperative heart failure and reduced left ventricular function after coronary artery bypass grafting (CABG).^7^, ^8^, ^9^ A post hoc analysis of the EXCEL trial showed that high baseline NT-proBNP levels in patients with left main disease undergoing revascularization were independently associated with long-term mortality.^10^ Furthermore, an association between NT-proBNP levels and mortality in patients with coronary artery disease and left ventricular dysfunction was demonstrated in a substudy of the STICH trial.^11^ A post hoc analysis of the PARADIGM study showed that patients achieving a significant decrease in NT-proBNP levels had lower rates of subsequent cardiovascular death or heart failure hospitalization regardless of their treatment group.^12^ However, prognostic implications of NT-proBNP dynamics prior to cardiac surgery in this vulnerable patient population remain unknown. Consequently, the objective of this study was therefore to explore the prognostic implications of preoperative NT-pro-BNP dynamics on 30-day and 5-year mortality after cardiac surgery.

## Methods

### Study population, data collection, and ethics approval

Data from 9,448 consecutive patients undergoing cardiac surgery between January 2010 and December 2020 at our tertiary reference university hospital (Innsbruck, Austria) were analyzed retrospectively. We excluded patients undergoing cardiac surgery without cardiopulmonary bypass (n = 906), patients <18 years old (n = 215), patients undergoing heart transplantation or implantation of a ventricular assist device or myxoma resection, and those with isolated aortic or grown-up congenital heart disease. Patients without preoperative NT-proBNP determination were also excluded from the analysis (n = 889), resulting in a final cohort of 6,938 patients for the analysis (Supplemental Figure 1). The indication for surgery was discussed within the HEART team, and a joint decision was taken by cardiac surgeons and cardiologists. The surgical strategy was decided by the surgeon performing the operation. General anesthesia was induced with midazolam, esketamine, propofol, fentanyl, and rocuronium after peripheral vein and radial arterial cannulation. Inhalation anesthesia (sevoflurane; 2%-2.5%, target minimum alveolar concentration: 0.8-1) and St. Thomas II cardioplegic solutions were used in all patients. NT-proBNP levels were routinely measured on the day before surgery. The same assay (Roche Diagnostics, Elecsys proBNP II) was used throughout the entire observation period.

Mortality data were obtained from the Austrian federal statistics institute (“*Statistik Austria*”). Follow-up data for the first 30 days after surgery were available for all patients; 3,137 patients did not survive until 5 years after follow-up or were lost to follow-up and were included with the maximum follow-up. Follow-up was censored after 5 years for analysis, resulting in a median follow-up time of 4.53 (2.00-5.00) years. This trial was performed in accordance with the Declaration of Helsinki, and permission to use anonymized data without patient consent for this study was obtained from the Innsbruck Medical University Institutional Review Board (UN 1350/2023).

### Validation cohort

Patients for whom preoperative NT-proBNP determinations were available (n = 3,117) were identified in the Swedish Cardiac Surgery Registry, which is part of the SWEDEHEART Registry.^13^^,^^14^ The study was approved by the Swedish Ethical Review Authority (registration number: 2021-00122). Details of the external validation cohort from the SWEDEHEART registry are provided in Supplemental Table 1.

### Evaluation of the effect of changes in NT-proBNP concentration before surgery

NT-proBNP levels determined at diagnosis of the underlying heart disease or assignment to surgery (median time before surgery: 56.0 [27.9-94.6] days) were available for 4,739 patients. These data were obtained from the referring cardiology departments. The same assay (Roche Diagnostics) was used for these determinations in 99.2% of patients, but as patients were referred from different departments, other tests were used in some cases. A detailed list of the assays used is presented in Supplemental Table 2.

### Study endpoints and statistical analysis

**Figure 1:**
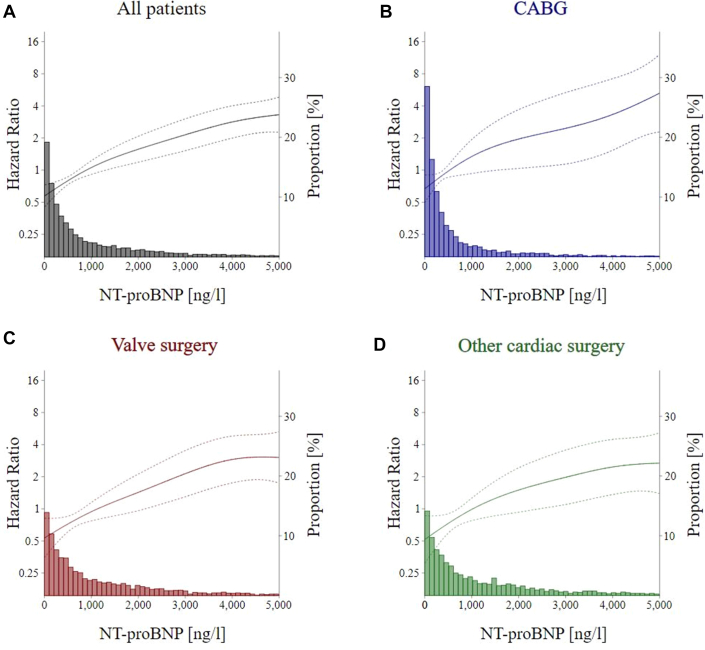
**Association Between Preoperative NT-proBNP Levels and 30-Day Mortality Cox Analysis With a Regression Spline Exploring the Relationship Between NT-proBNP Concentration and 30-Day Mortality, Adjusted for EuroSCORE II** Data for (A) all patients, (B) CABG patients, (C) patients undergoing valve surgery, and (D) patients undergoing combined or other surgery analyzed separately. CABG = coronary artery bypass grafting.

The primary outcomes were all-cause mortality at 30 days and 5 years after surgery. The secondary outcomes were the duration of stay in the intensive care unit (ICU), ultrafiltration, and the need for postoperative extracorporeal membrane oxygenation (ECMO). Patients were stratified into three groups according to the procedure performed: 1) isolated CABG surgery; 2) isolated valve procedures; and 3) combined CABG and valve procedures or other cardiac procedures. A detailed list of the procedures performed is presented in Supplemental Table 3. Continuous variables are presented as the median and interquartile range, whereas categorical variables are presented as frequencies and proportions. Differences between groups were assessed in Mann-Whitney *U* tests and chi-square tests, as appropriate. Time zero was defined as the time at which the surgical procedure was performed. We calculated the area under the receiver-operating characteristics curve on the plot of sensitivity vs specificity for NT-proBNP concentration for 30-day mortality after cardiac surgery. We performed multivariable Cox proportional hazards regression analysis to evaluate the association of NT-proBNP concentration with 30-day mortality, as previously described.^15^^,^^16^ We included NT-proBNP concentration in a penalized spline natural log-transformed covariate model with 2 degrees of freedom. The model was adjusted with EuroSCORE II as a continuous variable reflecting relevant preoperative comorbid conditions. The resulting predicted HR curves should be treated with caution, as they result from spline transformation, with all its inherent limitations and possible sources of error. Specifically, the fit of the model in the uppermost and lowest regions is not robust over the range of covariates or the number of spline curve knots.^15^^,^^16^ Splines are presented with 95% CIs. The estimates for the analyses in Figure 1 were obtained with R, version 4.1.2, with the splines package. The R script for the analyses is available from the authors upon reasonable request. Based on a previously defined clinically relevant threshold of 3,000 pg/mL in nonsurgical patients, this concentration cutoff was investigated in the Cox models.^17^^,^^18^ An NT-proBNP level of 3,000 ng/L was used to set the threshold separating the patients with low risk from those with high risk. A sensitivity analysis was performed using alternative NT-proBNP cutoffs of 1,000 ng/L and 2,000 ng/L to explore different thresholds for high-risk classification. Multivariable Cox proportional hazards regression and logistic regression analysis were performed to evaluate the association of the defined risk categories with outcomes after the procedure (duration of ICU stay, ECMO, ultrafiltration, 30-day and 5-year mortality). The proportional hazards assumption was assessed using log-minus-log survival plots for categorical predictors in the Cox regression models. Due to elevated NT-proBNP levels in patients undergoing hemodialysis, sensitivity analyses were performed excluding those on dialysis prior to surgery. Additional sensitivity analyses were conducted by stratifying the cohort based on the time interval between NT-proBNP measurement at the initial consultation and hospital admission (<30 days, 30-60 days, >60 days). All analyses were adjusted with EuroSCORE II as a covariable. HRs and ORs are reported with 95% CI. Follow-up was censored after 5 years for analysis. Patients for whom we did not have 5 years of follow-up (deceased or lost to follow-up) were included at their maximum follow-up. Kaplan-Meier estimates were used to plot survival curves, which were compared in log-rank tests. *P* values <0.05 were considered to indicate statistical significance. All statistical analyses were performed with SPSS Version 29.0 (IBM Corporation), MedCalc Version 20.216 (MedCalc Software Ltd), GraphPad Prism version 9.4.1 (Graph Pad Software Inc), and R 4.1.2 (The R Foundation for Statistical Computing).

## Results

### Characteristics of the patients and surgical interventions

We analyzed data for 6,938 consecutive patients undergoing cardiac surgery: 31.7% (2,201/6,938) were female, the median age was 69.4 years, 22.2% (1,539/6,938) had diabetes, 9.8% (677/6,938) suffered from chronic obstructive pulmonary disease, and 1.4% (78/5,691) were on long-term hemodialysis. A history of prior myocardial infarction was reported for 24.5% (1,700/6,938) of the patients, and preoperative left ventricular ejection fraction was 58% (50-63); 39.8% (2,312/5,808) of the cases were classified as NYHA II, and 42.4% (2,465/5,808) as New York Heart Association III (Table 1). Isolated CABG was performed in 34.8% (2,411/6,938) of the patients, 37.5% (2,603/6,938) underwent isolated valve procedures, and 27.7% (1,924/6,938) underwent other cardiac procedures (Supplemental Figure 1, Supplemental Table 4). Cardiac surgery was elective in 86.4% (5,899/6,824) of the patients and urgent in 11.6% (791/6,824), and 1.9% (134/6,824) of the patients underwent emergency or salvage procedures. The mean calculated EuroSCORE II was 2.1 (1.2-4.1), aortic cross-clamp time was 85 (65-111) minutes, and cardiopulmonary bypass time was 132 (104-172) minutes. Most patients spent <24 hours in the ICU after surgery (0 [0-2] days), and 11.1% (772/6,938) of the patients underwent postoperative renal ultrafiltration. Postcardiotomy ECMO was performed in 2.9% (203/6,938) of the patients, and 2.1% (149/6,938) of the patients died within the first 30 days after surgery (Table 2). The characteristics of the patients and the surgical procedures are presented in Supplemental Tables 4 and 5. The characteristics of the patients in the validation cohort and of the surgical procedures they underwent are presented in Supplemental Table 1.

**Table 1:** Patient Characteristics (N = 6,938)

Demographic characteristics	
Female	2,201 (31.7%)
Age (y)	69.4 [60.6-75.7]
BMI	26.0 [24.0-29.0]
Pre-existing conditions	
Diabetes	1,539 (22.2%)
Hypertension	5,770 (83.2%)
Dyslipidemia	5,152 (74.3%)
History of smoking	2,374 (34.2%)
COPD	677 (9.8%)
Creatinine (mg/dL)	0.98 [0.84-1.15]
Long-term dialysis	78 (1.4%)
Prior stroke	428 (6.2%)
Prior myocardial infarction	1,700 (24.5%)
LVEF (%)	58 [50-63]
LVEF grouped	
<20%	57 (0.8%)
21%-30%	198 (2.9%)
31%-50%	1,717 (25.0%)
>50%	4,895 (71.3%)
NYHA functional class	
I	736 (12.7%)
II	2,312 (39.8%)
III	2,465 (42.4%)
IV	295 (5.1%)
NT-proBNP (ng/L)	552 [208-1,591]

Values are median [IQR] or *n* (%).

BMI = body mass index; COPD = chronic obstructive pulmonary disease; LVEF = left ventricular ejection fraction; NT-proBNP = N-terminal prohormone of brain natriuretic peptide.

**Table 2:** Perioperative Characteristics (N = 6,938)

Surgical procedure	
Status	
Emergency or salvage	124 (1.9%)
Urgent	791 (11.6%)
Elective	5,899 (86.4%)
EuroSCORE II	2.1 [1.2-4.1]
Cross-clamp time (min)	85 [65-111]
Perfusion time (min)	132 [104-172]
Postoperative outcome	
Ultrafiltration	772 (11.1%)
Days in ICU	0 [0-2]
ECMO	203 (2.9%)
Death within 30 days	149 (2.1%)

Values are mean ± SD, median [IQR] or *n* (%)

ECMO = extracorporeal membrane oxygenation; ICU = intensive care unit.

### Association of preoperative NT-proBNP concentration with 30-day mortality

Median preoperative NT-proBNP concentration was 552 ng/L (208-1,591) (Table 1) for all patients, 307 ng/L (137-789) in patients undergoing isolated CABG, 764 ng/L (284-1,974) in patients undergoing isolated valve procedures, and 809 ng/L (287-2,066) in patients undergoing combined or other procedures (Supplemental Table 4). The area under the curve for the association of preoperative NT-proBNP concentration and 30-day mortality was 0.739 (95% CI: 0.728-0.749; *P* value <0.001) for all patients, 0.723 (95% CI: 0.704-0.740) for CABG patients, 0.721 (95% CI: 0.703-0.738) for patients undergoing valve procedures, and 0.758 (95% CI: 0.739-0.777) for patients undergoing combined or other surgery (Supplemental Figure 2). High preoperative levels of NT-proBNP were associated with a higher 30-day mortality in all patients. The same association was found when patients undergoing CABG, heart valve surgery, and combined or other cardiac procedures were analyzed separately (Figure 1). Metrics of the spline model are presented in Supplemental Table 6.

In the SWEDEHEART validation cohort, median preoperative NT-proBNP concentration was 393 ng/L (140-1,220) in all patients, 279 ng/L (120-640) in patients undergoing isolated CABG, 2,670 ng/L (2,330-3,225) in patients undergoing isolated valve procedures, and 6,990 ng/L (5,005-11,558) in patients undergoing combined or other procedures (Supplemental Table 1). The area under the curve of the receiver-operating characteristics curve for 30-day mortality was 0.830 (95% CI: 0.766-0.894, *P* < 0.001) for all patients, 0.770 (95% CI: 0.613-0.926; *P* = 0.001) for patients with CABG, 0.832 (95% CI: 0.747-0.917, *P* < 0.001) for patients with valve procedures, and 0.842 (95% CI: 0.711-0.973, *P* < 0.001) for patients undergoing combined or other procedures.

### High NT-proBNP levels are associated with impaired 30-day and 5-year mortality

**Figure 2:**
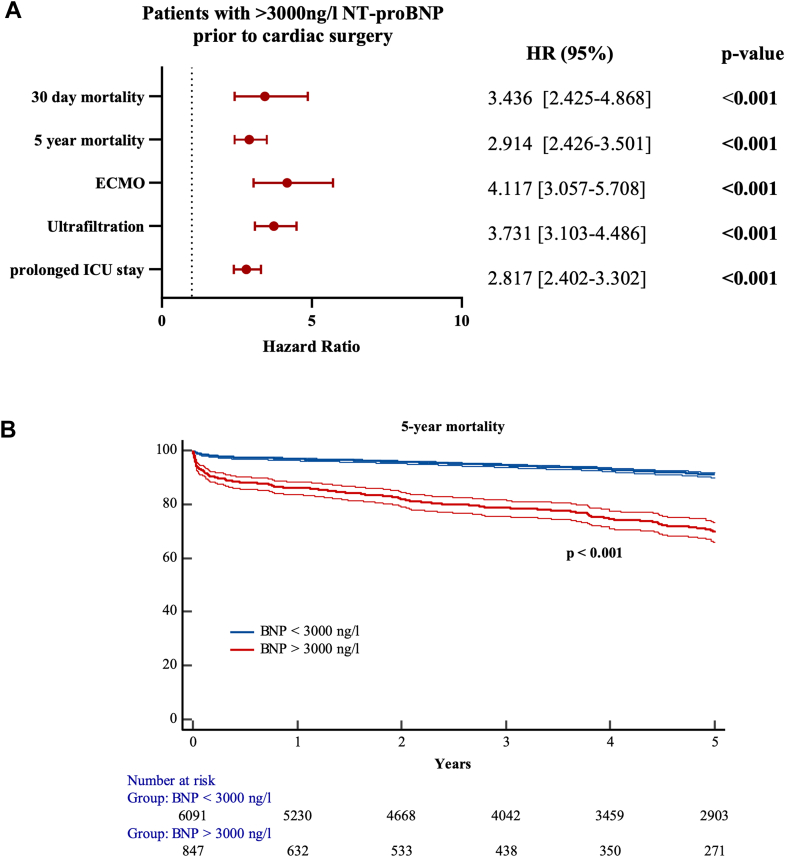
**Higher Preoperative NT-proBNP Levels Are Associated With Poor Postoperative Outcome** (A) Patients in the high-risk category were compared with patients in the low-risk category. Regression models were adjusted for EuroSCORE II. (B) Kaplan-Meier curves for 5-year mortality. CABG = coronary artery bypass grafting; ECMO = extracorporeal membrane oxygenation; ICU = intensive care unit, prolonged ICU stay = longer than 1 day.

A threshold of 3,000 pg/mL was defined as to identify high-risk patients as suggested previously in nonsurgical patients.^17^^,^^18^ Overall, 847 patients exhibited preoperative NT-proBNP levels >3,000 ng/L (12.2%). High-risk patients had a significantly longer stay in the ICU, higher rates of renal ultrafiltration, higher rates of ECMO, and a greater 30-day mortality (all *P* < 0.001) (Table 3). Patients with higher preoperative NT-proBNP concentrations were markedly sicker (Supplemental Table 7). We therefore performed a Cox regression proportional hazards model analysis adjusted for EuroSCORE II, major comorbid conditions, such as renal or ventricular dysfunction, age, urgency, and type of surgery. Patients in the high-risk category had a greater risk of prolonged ICU stay (OR: 2.82; 95% CI: 2.40-3.30), ultrafiltration (OR: 3.73; 95% CI: 3.10-4.49), or postcardiotomy ECMO (OR: 4.12; 95% CI: 3.06-5.71) and a greater 30-day mortality (HR: 3.44; 95% CI: 2.43-4.89) and 5-year mortality (HR: 2.91; 95% CI: 2.43-3.50; *P* for all patients <0.001). Five-year survival was lower for the higher risk category (log rank: *P* value <0.001), regardless of the type of surgery performed (Figure 2, Supplemental Figure 3). Sensitivity analyses using alternative thresholds (1,000 and 2,000 ng/L) identified high-risk subgroups with significantly worse outcomes; however, these cutoffs classified 36% and 20% of the cohort as high risk, respectively, which exceeded the intended specificity of the study’s risk-stratification approach.

**Table 3:** Risk Categories Based on Preoperative NT-proBNP Levels

	Low Risk <3,000 ng/L	High Risk >3,000 ng/L	*P* Value
CABG			
ICU days	0 [0-1]	4.5 [1-9]	<0.001
Ultrafiltration	134 (5.9%)	51 (24.9%)	<0.001
ECMO	28 (1.2%)	15 (10.3%)	<0.001
30-day mortality	30 (1.3%)	10 (6.8%)	<0.001
n (%)	2,265 (93.9%)	146 (6.1%)	
Valve(s)			
ICU days	0 [0-1]	2 [0-6]	<0.001
Ultrafiltration	179 (8.1%)	107 (28.2%)	<0.001
ECMO	41 (1.8%)	29 (7.6%)	<0.001
30-day mortality	34 (1.5%)	26 (6.8%)	<0.001
n (%)	2,223 (85.4%)	380 (14.6%)	
Other procedures			
ICU days	1 [0-3]	4 [0-11.5]	<0.001
Ultrafiltration	186 (11.6%)	115 (35.8%)	<0.001
ECMO	43 (2.7%)	47 (14.6%)	<0.001
30-day mortality	23 (1.4%)	26 (8.1%)	<0.001
n (%)	1,603 (83.3%)	321 (16.7%)	

Low risk <3,000 ng/L; high risk >3,000 ng/L.

CABG = coronary artery bypass grafting; ECMO = extracorporeal membrane oxygenation; ICU = intensive care unit; NT-proBNP = N-terminal prohormone of brain natriuretic peptide.

Exclusion of patients on dialysis prior to surgery in sensitivity analyses produced comparable results (Supplemental Tables 8 and 9).

### Reduction of preoperative NT-proBNP concentration improves 30-day and 5-year survival

**Figure 3:**
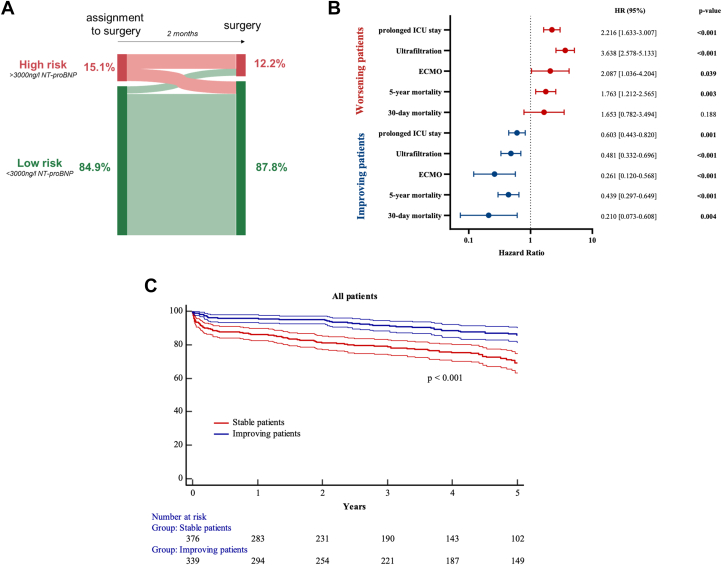
**Preoperative Changes in NT-proBNP Concentration** (A) NT-proBNP levels measured at the time of assignment to surgery and just before surgery were used to assign patients to risk categories. (B) Patients in the low-risk group who experienced a worsening of their risk category between the 2 evaluations were compared with those who remained in the low-risk group. Similarly, patients in the high-risk group whose risk category improved between the evaluations were compared with those who remained in the high-risk group. Regression models were adjusted for EuroSCORE II. (C) Kaplan-Meier curves demonstrated lower 5-year mortality in patients whose risk category improved between the 2 preoperative evaluations. ECMO = extracorporeal membrane oxygenation; ICU = intensive care unit, prolonged ICU stay = longer than 1 day.

We assessed the effect of preoperative changes in NT-proBNP levels on 30-day mortality by analyzing NT-proBNP levels obtained at diagnosis of the underlying heart disease or assignment to surgery and NT-proBNP levels on the day before surgery. NT-proBNP concentrations at initial consultation were available for 4,739 patients (median time before surgery: 56.0 [27.9-94.6] days; 27.4% <30 days; 26.3% = 30-60 days; 46.3% > 60 days). At the initial consultation, 15.1% of patients had NT-proBNP concentrations >3,000 ng/L (high-risk category), and 84.9% had NT-proBNP concentrations <3,000 ng/L (low-risk category). Between this initial consultation and surgery, 7.2% of patients presented an improvement, passing from the high-risk category into the low-risk category, and 4.3% presented a deterioration, passing from the low-risk group into the high-risk category (Figure 3A, Supplemental Table 10). Patients initially assigned to the low-risk category presenting a deterioration in NT-proBNP-based risk category by the time of surgery had poor postoperative outcomes, spending longer time in the ICU (2 vs 0 days; *P* < 0.001), with higher rates of ultrafiltration (32.5% vs 7.9%; *P* < 0.001) and ECMO use (6.8% vs 1.7%; *P* < 0.001) and a greater 30-day mortality (4.4% vs 1.5%; *P* value = 0.002) (Table 4). After adjustment of the regression model for EuroSCORE II, such patients were found to have a higher risk of prolonged ICU stay (OR: 2.22; 95% CI: 1.63-3.01; *P* < 0.001), ultrafiltration (OR: 3.64; 95% CI: 2.58-5.13; *P* < 0.001), ECMO (OR: 2.09; 95% CI: 1.04-4.20; *P* = 0.039), and higher 5-year mortality rates (HR: 1.76; 95% CI: 1.21-2.57; *P* = 0.003) (Figure 3B).

**Table 4:** Effect of Changes in Risk Category Before Surgery

	Improvement (n = 339)	Stable in High-Risk Category (n = 376)	*P* Value
ICU days	1 [0-5]	3 [0-8]	<0.001
Prolonged ICU stay	148 (43.7%)	224 (59.6%)	<0.001
Ultrafiltration	57 (16.8%)	122 (32.4%)	<0.001
ECMO	9 (2.7%)	40 (10.6%)	<0.001
30-day Mortality	4 (1.2%)	25 (6.6%)	<0.001

	**Deterioration (n = 206)**	**Stable in Low-Risk Category (n = 3,818)**	

ICU days	2 [0-7.25]	0 [0-1]	<0.001
Prolonged ICU stay	108 (52.4%)	944 (24.7%)	<0.001
Ultrafiltration	67 (32.5%)	301 (7.9%)	<0.001
ECMO	14 (6.8%)	65 (1.7%)	<0.001
30-day mortality	9 (4.4%)	59 (1.5%)	0.002

Patients initially classified as high risk at evaluation, who showed improvement in their risk category before surgery, were compared with those whose risk level remained unchanged. Similarly, patients classified as low risk at the initial evaluation, who experienced an increase in risk level before surgery, were compared with those who remained in the low-risk category. This analysis was limited to patients for whom NT-proBNP concentrations were measured at the time of diagnosis of the underlying heart disease or assignment to surgery (n = 4,739).

ECMO = extracorporeal membrane oxygenation; ICU = intensive care unit, prolonged ICU stay = longer than 1 day.

Conversely, patients initially assigned to the high-risk category who displayed an improvement in risk category by the time of surgery had better perioperative outcomes than patients who remained in the same risk category, with shorter stays in the ICU (1 vs 3 days; *P* < 0.001), less frequent use of ultrafiltration (16.8 vs 32.4%; *P* < 0.001) or ECMO (2.7 vs 10.6%; *P* < 0.001), and a lower 30-day mortality (1.2 vs 6.6%; *P* < 0.001) (Table 4). Following adjustment of the regression model for EuroSCORE II, such patients were found to have shorter stays in the ICU (OR: 0.60 [0.44-0.82]), less frequent use of ultrafiltration (OR: 0.48 [0.33-0.70]) or ECMO (OR: 0.26 [0.12-0.57]; all *P* < 0.001), and a lower 30-day mortality (HR: 0.21 [0.07-0.61]; *P* = 0.004) (Figure 3B). These findings were supported by Kaplan-Meier estimates, which revealed higher 5-year survival rates in patients ameliorating preoperative NT-proBNP levels to <3,000 ng/L (log-rank: *P* < 0.001, HR: 0.44 [0.30-0.65]; *P* < 0.001) (Figure 3C).

Sensitivity analyses based on the time interval between NT-proBNP measurement at the initial consultation and hospital admission demonstrated that the overall trends in biomarker dynamics and their associations with outcomes remained broadly consistent across groups. These findings suggest that the prognostic value of NT-proBNP change is robust to moderate variability in the timing of the initial measurement (Supplemental Tables 11 to 13).

## Discussion

The key findings of this study analyzing 6,938 consecutive patients undergoing cardiac surgery were:

1) NT-proBNP levels were associated with 30-day and 5-year mortality after adjustment for EuroSCORE II;
2) Patients with preoperative NT-proBNP levels >3,000 ng/L exhibited longer ICU stays, required postoperative ultrafiltration or ECMO more frequently, and had impaired 30-day and 5-year survival;
3) Improvements of preoperative NT-proBNP <3,000 ng/L were associated with lower rates of ECMO, ultrafiltration, reduced ICU stay, and better 30-day and 5-year survival (Central Illustration).

**Central Illustration:**
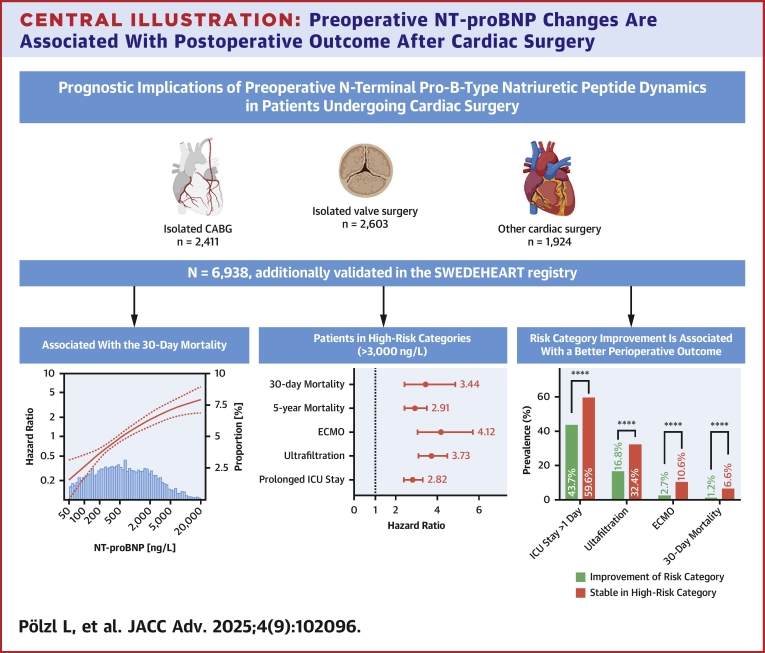
**Preoperative NT-proBNP Changes Are Associated With Postoperative Outcome After Cardiac Surgery** A consecutive cohort of 6,938 patients undergoing cardiac surgery was analyzed. Preoperative levels of NT-proBNP are significantly associated with 30-day postoperative mortality. Patients with NT-proBNP concentrations exceeding 3,000 ng/L are at markedly increased risk of both short- and long-term adverse outcomes following cardiac surgery. Conversely, preoperative reduction of NT-proBNP levels to below 3,000 ng/L correlates with improved survival, including lower 30-day and 5-year mortality rates. These findings underscore the prognostic value of NT-proBNP as a biomarker for risk stratification and potential therapeutic optimization prior to cardiac surgical interventions. This image was created with BioRender.com.

Our findings suggest that at least patients with NT-proBNP concentrations >3,000 ng/L should undergo a preoperative optimization of medical treatment to improve perioperative outcomes.^17^^,^^18^

Previous studies have linked preoperative NT-proBNP levels to survival in patients undergoing CABG and patients with ventricular dysfunction.^7^, ^8^, ^9^ A post hoc analysis of the EXCEL trial revealed an association of outcome with risk-adjusted preoperative NT-proBNP levels, regardless of the type of revascularization.^10^ Our study confirms this correlation, regardless of the type of cardiac surgery. NT-proBNP concentration remained an independent predictor of outcome even after adjustment for EuroSCORE II, including left ventricular function. Thus, preoperative NT-proBNP determinations are a useful addition to risk assessments in patients undergoing cardiac surgery, for patients both with and without obvious symptoms of heart failure. There is clear evidence that preoperative NT-proBNP levels are a strong predictor of cardiovascular events in the first 30 days after surgery in patients undergoing noncardiac surgery.^19^ In our study, NT-proBNP concentration was found to be an independent predictor of all-cause mortality at both 30 days and 5 years.

We then investigated the association between changes in NT-proBNP levels before surgery and postoperative outcomes. We compared NT-proBNP concentrations at the time of diagnosis of the underlying heart disease or at the time at which the decision to perform surgery was made with those obtained 1 day before the surgery. Preoperative improvements in NT-proBNP-based risk category were clearly associated with a lower risk of perioperative complications and lower mortality at 30 days and 5 years. These findings are consistent with those of previous studies demonstrating beneficial effects of decreasing BNP concentration on cardiovascular events in patients with heart failure.^12^^,^^20^

Our findings suggest that at least patients with NT-proBNP concentrations >3,000 ng/L should undergo a preoperative optimization of medical treatment by heart failure specialists to improve their perioperative outcomes. Further risk stratification within the intermediate NT-proBNP range could enhance clinical value, highlighting the need for future prospective studies to refine NT-proBNP-based risk thresholds in surgical populations. In addition, prospective studies are required to determine whether active efforts to decrease preoperative NT-proBNP levels actually lead to an improvement in perioperative and postoperative outcomes. It also remains to be determined which patients gain the most benefit from decreases in preoperative NT-proBNP concentration, since the absence of a detailed echocardiographic assessment of diastolic function limits the ability to fully differentiate between pathophysiological states such as diastolic dysfunction or volume overload. Nevertheless, our findings indicate that the effect of passing into a lower NT-proBNP risk category is not directly dependent on surgical procedure, being instead associated with preoperative patient characteristics. Clinical programs focusing on the preoperative optimization of patients undergoing cardiac surgery may therefore have benefits for both short- and long-term outcomes.

According to current guidelines, all patients, including those with heart failure, should be on guideline-directed medical therapy (GDMT) before surgery.^21^ The EXCEL trial reported a clear improvement of clinical outcomes in patients on GDMT undergoing PCI or CABG.^10^ However, this trial also revealed that only 29% of the patients were on GDMT before the revascularization procedure.^22^ These figures probably reflect the clinical reality. Our data should therefore strongly encourage clinicians to enforce preoperative GDMT in all patients, including measuring preoperative NT-proBNP levels, with the aim of improving outcomes after cardiac surgery. Importantly, while our findings suggest an association between preoperative NT-proBNP reduction and improved outcomes, the mechanisms underlying this improvement remain unclear. Given the retrospective design of our study and lack of detailed data on preoperative medical interventions, causality cannot be inferred. Prospective studies are needed to confirm these observations and clarify whether targeted preoperative optimization can directly influence biomarker levels and clinical outcomes.

### Strengths and limitations

This study has several strengths, including a large study cohort of consecutive patients and detailed information about the characteristics of the patients and the cardiac surgery they underwent. The results were validated in an external study cohort. The limitations of this study include its observational single-center study design, with all the limitations such studies entail, including residual confounding and bias. Furthermore, the GDMT changed during the observation period, which limits the comparability of the patients. Moreover, no data were available on glycemic control or the specific use and optimization of GDMT, which further limits our ability to assess the impact of preoperative medical management on outcomes. Similarly, the lack of invasive hemodynamic data (central venous pressure, pulmonary capillary wedge pressure, and cardiac output) limits our ability to interpret the mechanistic significance of elevated NT-proBNP levels, particularly in relation to volume status and ventricular function during the perioperative period. The analysis of NT-proBNP levels should be interpreted with caution. Due to the retrospective nature of our study, NT-proBNP levels for the initial consultation were missing for several patients. In addition, the time interval between baseline NT-proBNP measurement and surgery varied widely across the cohort, potentially introducing variability due to interim clinical fluctuations. While sensitivity analyses suggest consistency of results across timing subgroups, this remains an inherent limitation of retrospective data.

## Conclusions

A reduction of NT-proBNP levels below 3,000 ng/L before surgery was associated with lower 30-day and 5-year mortality after cardiac surgery. Patients with high NT-proBNP concentrations may benefit from preoperative optimization to lower their NT-proBNP and improve perioperative outcomes.

Perspectives **COMPETENCY IN MEDICAL KNOWLEDGE:** Preoperative NT-proBNP levels are independently associated with 30-day and 5-year mortality after cardiac surgery.

**COMPETENCY IN PATIENT CARE:** Patients undergoing cardiac surgery with increased NT-proBNP levels should be made aware that a decrease of their NT-proBNP prior to surgery has a beneficial impact on the perioperative outcome.

**TRANSLATIONAL OUTLOOK 1:** Within this retrospective study, a decrease in 30-day and 5-year mortality after cardiac surgery was observed in patients with decreasing NT-proBNP levels before surgery. Further prospective studies are required to determine whether active efforts to decrease preoperative NT-proBNP levels actually lead to an improvement in perioperative and postoperative outcomes.

**TRANSLATIONAL OUTLOOK 2:** Our data strongly encourage clinicians to enforce preoperative guideline-directed medical therapy in all patients, with the aim of improving outcomes after cardiac surgery.

### Data availability statement

The data supporting this article will be shared by the corresponding author on reasonable request.

## Funding support and author disclosures

Dr Jeppsson has received fees for consultancy or speaker’s fees from AstraZeneca, Werfen, 10.13039/100004326Bayer, Novo Nordisk, and LFB Biotechnologies, all unrelated to this work. All other authors have reported that they have no relationships relevant to the contents of this paper to disclose.
