# Supplementary material for: Prognostic Implications of Preoperative N-Terminal Pro-B-Type Natriuretic Peptide Dynamics in Patients Undergoing Cardiac Surgery
Source: JACC Adv. 2025 Aug 26;4(9):102096. doi: 10.1016/j.jacadv.2025.102096 (PMC12409294; doi:10.1016/j.jacadv.2025.102096)
Supplement: Supplemental Material [file mmc1.docx]

**Supplemental Table 1:** Baseline and surgical characteristics of the external validation cohort

|  | **Patients from the SWEDEHEART registry  *n* = 3117** |
| --- | --- |
| Procedure |  |
| Isolated CABG | 1620 (52.0%) |
| Isolated valve surgery | 1184 (38.0%) |
| Other cardiac surgery | 313 (10.0%) |
| **Demographic characteristics** |  |
| Sex (female) | 685 (22.0%) |
| Age (years) | 70 [62-75] |
| BMI | 26.9 [24.4-30.1] |
| **Pre-existing conditions** |  |
| Diabetes | 802 (25.7%) |
| Hypertension | 2002 (64.2%) |
| Dyslipidemia | 976 (31.3%) |
| COPD | 347 (11.1%) |
| Serum creatinine (mg/dl) | 0.93 [0.81-1.09] |
| Long-term dialysis | 29 (0.9%) |
| Prior stroke | 185 (5.9%) |
| Prior myocardial infarction | 961 (30.8%) |
| LV-EF (%) |  |
| <20% | 35 (1.1%) |
| 21-30% | 113 (3.6%) |
| 31-50% | 775 (24.9%) |
| >50% | 2197 (70.4%) |
| NYHA class |  |
| *I* | 380 (12.2%) |
| *II* | 1252 (40.2%) |
| *III* | 1250 (40.1%) |
| *IV* | 212 (6.8%) |
| NT-proBNP (ng/l) | 393 [140-1220] |
| **Surgical procedure** |  |
| *Emergency* | 141 (4.8%) |
| *Urgent* | 1268 (40.7%) |
| *Elective* | 1698 (54.5%) |
| EuroSCORE II | 1.65 [1.06-2.98] |
| Cross-clamp time (min) | 64 [47-86] |
| Perfusion time (min) | 93 [73-122] |
| **Postoperative course** |  |
| Ultrafiltration | 49 (1.6%) |
| Death within 30 days | 41 (1.3%) |
| Values are expressed as the mean ± SD, median [interquartile range] or *n* (%) | |

BMI = body mass index; CABG = coronary artery bypass grafting; COPD = chronic obstructive pulmonary disease; LV-EF = left ventricular ejection fraction; NT-proBNP = N-terminal prohormone of brain natriuretic peptide, NYHA = New York Heart Association.

**Supplemental Table 2:** NT-proBNP assays used

| **NT-proBNP assays used** | | | | | |
| --- | --- | --- | --- | --- | --- |
| **At initial consultation or assignment to surgery** *n = 4739* | | | **One day before surgery** *n = 6938* | | |
| Roche | 4644 | 98.00% | Roche | 6938 | 100% |
| Siemens | 71 | 1.50% |  |  |  |
| Abbott | 24 | 0.51% |  |  |  |

**Supplemental Table 3:** Detailed list of procedures for the “other procedures” subgroup.

| **Procedure** | ***n* (%)** |
| --- | --- |
| Isolated aortic root procedure | 132 (6.8%) |
| AVR+replacement of the ascending aorta | 169 (8.8%) |
| CABG+valve procedures | 959 (49.8%) |
| CABG+AVR+replacement of the ascending aorta | 59 (3.1%) |
| AVR+reduction of the ascending aorta | 65 (3.4%) |
| AVR+myectomy | 35 (1.8%) |
| AVR+replacement of the ascending aorta and part of the aortic arch | 20 (1.0%) |
| Bentall+MVR | 4 (0.2%) |
| Bentall+CABG | 24 (1.2%) |
| CABG+AVR+reduction of the ascending aorta | 20 (1.0%) |
| Bentall+CABG+replacement of the ascending aorta and part of the aortic arch | 4 (0.2%) |
| Bentall+CABG+replacement of the ascending aorta | 5 (0.3%) |
| AVR+CABG+myectomy | 9 (0.5%) |
| Bentall+partial replacement of the aortic arch | 36 (1.9%) |
| CABG+Dor procedure | 26 (1.4%) |
| CABG+replacement of the ascending aorta | 22 (1.1%) |
| Other | 335 (17.4%) |
|  |  |

AVR = aortic valve replacement; CABG = coronary artery bypass grafting; MVR = mitral valve repair.

**Supplemental Table 4:** Patient characteristics by type of surgery

|  | **Isolated CABG *n* = 2411 (34.8%)** | **Isolated valve surgery *n* = 2603 (37.5%)** | **Other cardiac surgery *n* = 1924 (27.7%)** |
| --- | --- | --- | --- |
| **Demographic characteristics** |  |  |  |
| Sex (female) | 425 (17.6%) | 1190 (45.7%) | 586 (30.5%) |
| Age (years) | 68.5 [60.6-74.5] | 69.4 [59.7-76.4] | 70.9 [62.1-76.2] |
| BMI | 27.0 [24.5-30.0] | 25.7 [23.0-29.0] | 26.0 [24.0-29.0] |
| **Pre-existing conditions** |  |  |  |
| Diabetes | 779 (32.3%) | 363 (13.9%) | 397 (20.6%) |
| Hypertension | 2244 (93.1%) | 1919 (73.7%) | 1607 (83.5%) |
| Dyslipidemia | 2225 (92.3%) | 1526 (58.6%) | 1401 (72.8%) |
| History of smoking | 1150 (47.7%) | 599 (23.0%) | 625 (32.5%) |
| COPD | 212 (8.8%) | 265 (10.2%) | 200 (10.4%) |
| Creatinine (mg/dl) | 0.98 [0.85-1.14] | 0.96 [0.83-1.13] | 1.01 [0.85-1.18] |
| Long-term dialysis | 29 (1.5%) | 25 (1.1%) | 24 (1.5%) |
| Prior stroke | 145 (6.0%) | 152 (5.8%) | 131 (6.8%) |
| Prior myocardial infarction | 1198 (49.7%) | 118 (4.5%) | 384 (20.0%) |
| LV-EF (%) | 57 [48-62] | 60 [51-64] | 56 [47-62] |
| LV-EF grouped |  |  |  |
| *<20%* | 21 (0.9%) | 21 (0.8%) | 15 (0.8%) |
| *21-30%* | 69 (2.9%) | 50 (1.9%) | 79 (4.2%) |
| *31-50%* | 655 (27.4%) | 540 (20.9%) | 522 (27.5%) |
| *>50%* | 1644 (68.8%) | 1968 (76.3%) | 1283 (67.6%) |
| NYHA class |  |  |  |
| *I* | 320 (15.8%) | 214 (9.8%) | 202 (12.6%) |
| *II* | 914 (45.2%) | 796 (36.4%) | 602 (37.6%) |
| *III* | 690 (34.1%) | 1083 (49.5%) | 692 (43.3%) |
| *IV* | 97 (4.8%) | 95 (4.3%) | 103 (6.4%) |
| NT-proBNP (ng/l) | 307 [137-789] | 764 [284-1974] | 809 [287-2066] |
| Values are expressed as the median [interquartile range] or *n* (%) | | | |

BMI = body mass index; CABG = coronary artery bypass grafting; COPD = chronic obstructive pulmonary disease; LV-EF = left ventricular ejection fraction; NT-proBNP = N-terminal prohormone of brain natriuretic peptide, NYHA = New York Heart Association.

**Supplemental Table 5:** Perioperative characteristics by type of surgery

|  | **Isolated CABG *n* = 2411 (34.8%)** | **Isolated valve surgery *n* = 2603 (37.5%)** | **Other cardiac surgery *n* = 1924 (27.7%)** |
| --- | --- | --- | --- |
| **Surgical procedure** |  |  |  |
| Status |  |  |  |
| *Salvage* | 6 (0.3%) | 4 (0.2%) | 5 (0.3%) |
| *Emergency* | 61 (2.6%) | 14 (0.5%) | 44 (2.3%) |
| *Urgent* | 462 (19.6%) | 158 (6.1%) | 171 (9.0%) |
| *Elective* | 1829 (77.6%) | 2398 (93.2%) | 1672 (88.4%) |
| EuroSCORE II | 1.4 ± 2.9 | 1.9 ± 5.4 | 3.7 ± 8.1 |
| Cross-clamp time (min) | 68 [54-83] | 89 [70-114] | 109 [87-135] |
| Perfusion time (min) | 111 [91-132] | 140 [108-185] | 161 [130-202] |
| **Postoperative course** |  |  |  |
| Ultrafiltration | 185 (7.7%) | 286 (11.0%) | 301 (15.6%) |
| Days in the ICU | 0 [0-2] | 0 [0-2] | 1 [0-4] |
| ECMO | 43 (1.8%) | 70 (2.7%) | 90 (4.7%) |
| Death within 30 days | 40 (1.7%) | 60 (2.3%) | 49 (2.5%) |
| Values are expressed as the mean ± SD, median [interquartile range] or *n* (%) | | | |

CABG = coronary artery bypass grafting; ECMO = extracorporeal membrane oxygenation; ICU = intensive care unit.

**Supplemental Table 6:** Metrics of spline model.

|  | **coef** | **se(coef)** | **se2** | **Chisq** | **DF** | **p-value** |
| --- | --- | --- | --- | --- | --- | --- |
| EuroSCORE II | 0.05215 | 0.005283 | 0.005266 | 97.42 | 1.00 | 5.6e-23 |
| pspline(BNP_ln, 2), linea | 0.43643 | 0.065597 | 0.065597 | 44.26 | 1.00 | 2.9e-23 |
| pspline(BNP_ln, 2), nonli | |  |  | 2.90 | 1.07 | 9.7e-02 |
|  |  |  |  |  |  |  |
|  | **exp(coef)** | **exp(-coef)** | **lower .95** | **upper .95** |  |  |
| EuroSCORE II | 1.054 | 0.949191 | 1.0427 | 1.064 |  |  |
| ps(BNP_ln)3 | 3.179 | 0.314526 | 0.7537 | 13.413 |  |  |
| ps(BNP_ln)4 | 10.098 | 0.099031 | 1.0029 | 101.670 |  |  |
| ps(BNP_ln)5 | 29.274 | 0.034160 | 2.2205 | 385.929 |  |  |
| ps(BNP_ln)6 | 61.328 | 0.016306 | 4.8178 | 780.665 |  |  |
| ps(BNP_ln)7 | 101.100 | 0.009891 | 8.0312 | 1272.687 |  |  |
| ps(BNP_ln)8 | 136.941 | 0.007302 | 9.7441 | 1924.514 |  |  |
| ps(BNP_ln)9 | 182.140 | 0.005490 | 8.0620 | 4115.002 |  |  |

**Supplemental Table 7:** Baseline characteristics for patients in different risk categories.

|  | **Low risk n = 6091 (87.8%)** | **High risk n = 847 (12.2%)** | **p-value** |
| --- | --- | --- | --- |
| **Demographic characteristics** |  |  |  |
| Sex (female) | 1895 (31.1%) | 306 (36.1%) | **0.003** |
| Age (years) | 68.8 [60.0-75.3] | 72.9 [65.9-77.6] | **<0.001** |
| BMI | 26.0 [24.0-29.0] | 25.0 [23.0-28.0] | **<0.001** |
| **Pre-existing conditions** |  |  |  |
| Diabetes | 1292 (21.2%) | 247 (29.2%) | **<0.001** |
| Hypertension | 5053 (83.0%) | 717 (84.7%) | 0.217 |
| Dyslipidemia | 4550 (74.7%) | 602 (71.1%) | **0.024** |
| History of smoking | 2090 (34.3%) | 284 (33.5%) | 0.653 |
| COPD | 535 (8.8%) | 142 (16.8%) | **<0.001** |
| Creatinin (mg/dl) | 0.96 [0.83-1.12] | 1.17 [0.96-1.53] | **<0.001** |
| Long-term dialysis | 23 (0.5%) | 55 (7.3%) | **<0.001** |
| Prior stroke | 350 (5.7%) | 78 (9.2%) | **<0.001** |
| Prior myocardial infarction | 1449 (23.8%) | 251 (29.6%) | **<0.001** |
| LV-EF (%) | 60 [51-64] | 47 [37-58] | **<0.001** |
| LV-EF grouped |  |  | **<0.001** |
| *<20%* | 23 (0.4%) | 34 (4.1%) |  |
| *21-30%* | 133 (2.2%) | 65 (7.8%) |  |
| *31-50%* | 1320 (21.9%) | 397 (47.4%) |  |
| *>50%* | 4554 (75.5%) | 341 (40.7%) |  |
| NYHA class |  |  | **<0.001** |
| *I* | 713 (13.9%) | 23 (3.3%) |  |
| *II* | 2168 (42.4%) | 144 (20.8%) |  |
| *III* | 2079 (40.6%) | 386 (55.9%) |  |
| *IV* | 157 (3.1%) | 138 (20.0%) |  |
| NT-proBNP (ng/l) | 441 [181-1077] | 5261 [3810-8614] | **<0.001** |
| Values are mean ± SD, median [interquartile range] or n (%) |  |  |  |
|  |  |  |  |

BMI = body mass index; COPD = chronic obstructive pulmonary disease; LV-EF = left ventricular ejection fraction; NT-proBNP = N-terminal prohormone of brain natriuretic peptide, NYHA = New York Heart Association.

**Supplemental Table 8:** Regression models excluding dialysis patients.

|  | **HR** | **p-value** |
| --- | --- | --- |
| 30-day mortality | 3.623 [2.482-5.289] | **<0.001** |
| Five-year mortality | 2.916 [2.374-3.582] | **<0.001** |
| ECMO | 3.800 [2.718-5.313] | **<0.001** |
| Ultrafiltration | 3.867 [3.171-4.717] | **<0.001** |
| prolonged ICU stay | 2.662 [2.233-3.175] | **<0.001** |
| adjusted to ESII | | |

ECMO = extracorporeal membrane oxygenation; ESII = EuroSCORE II; HR = Hazard Ratio; ICU = intensive care unit.

**Supplemental Table 9:** Outcome for low- and high-risk patients excluding dialysis patients.

|  | Low Risk | High risk | p-value |
| --- | --- | --- | --- |
|  | < 3000 ng/l | > 3000 ng/l |  |
| Prolonged ICU stay | 1308 (26.6%) | 411 (59.1%) | **<0.001** |
| Ultrafiltration | 435 (8.8%) | 242 (34.8%) | **<0.001** |
| ECMO | 102 (2.1%) | 76 (10.9%) | **<0.001** |
| 30-day mortality | 71 (1.4%) | 52 (7.5%) | **<0.001** |
| N | 4917 (87.6%) | 696 (12.4%) |  |

ECMO = extracorporeal membrane oxygenation; ICU = intensive care unit.

**Supplemental Table 10:** Preoperative changes in risk category

|  | **Risk group before admission** | | |
| --- | --- | --- | --- |
| **Risk group at surgery** |  | Low | High |
|  | Low | 3818 (80.6%) | 339 (7.2%) |
|  | High | 206 (4.3%) | 376 (7.9%) |

**Supplemental Table 11:** Effect of changes in risk category before surgery; time between the baseline NT-proBNP measurement and surgery less than 30 days.

| **Time of dynamic: <30d** | **Improvement** (n = 86)*^1^* | **Stable in high-risk category** (n = 125)*^1^* | **p-value***^2^* |
| --- | --- | --- | --- |
| ICU stay (days) | 1 (0, 4) | 4 (1, 11) | **<0.001** |
| ICU days >1 | 35 (41%) | 82 (66%) | **<0.001** |
| ECMO | 2 (2.3%) | 25 (20%) | **<0.001** |
| Ultrafiltration | 16 (19%) | 51 (41%) | **<0.001** |
| 30-day mortality | 1 (1.2%) | 13 (10%) | **0.008** |
| *^1^* Median (Q1, Q3); n (%) | | | |
| *^2^* Wilcoxon rank sum test; Pearson’s Chi-squared test | | | |
| **Time of dynamic: <30d** | **Deterioration** (n = 44)*^1^* | **Stable in low-risk category** (n = 1043)*^1^* | **p-value***^2^* |
| ICU stay (days) | 1.5 (0.0, 8.0) | 0.0 (0.0, 1.0) | **<0.001** |
| ICU days >1 | 22 (50%) | 239 (23%) | **<0.001** |
| ECMO | 3 (6.8%) | 14 (1.3%) | **0.028** |
| Ultrafiltration | 14 (32%) | 66 (6.3%) | **<0.001** |
| 30-day mortality | 1 (2.3%) | 15 (1.4%) | 0.5 |
| *^1^* Median (Q1, Q3); n (%) | | | |
| *^2^* Wilcoxon rank sum test; Pearson’s Chi-squared test; Fisher’s exact test | | | |

ECMO = extracorporeal membrane oxygenation; ICU = intensive care unit.

**Supplemental Table 12:** Effect of changes in risk category before surgery; time between the baseline NT-proBNP measurement and surgery 30 to 60 days.

| **Time of dynamic: 30-60d** | **Improvement** (n = 73)*^1^* | **Stable in high-risk category** (n = 109)*^1^* | **p-value***^2^* |
| --- | --- | --- | --- |
| ICU stay (days) | 1.0 (0.0, 4.0) | 2.0 (0.0, 8.0) | 0.086 |
| ICU days >1 | 33 (45%) | 63 (58%) | 0.10 |
| ECMO | 3 (4.1%) | 9 (8.3%) | 0.4 |
| Ultrafiltration | 8 (11%) | 30 (28%) | **0.007** |
| 30-day mortality | 0 (0%) | 6 (5.5%) | 0.083 |
| *^1^* Median (Q1, Q3); n (%) | | | |
| *^2^* Wilcoxon rank sum test; Pearson’s Chi-squared test; Fisher’s exact test | | | |
| **Time of dynamic: 30-60d** | **Deterioration** (n = 41)*^1^* | **Stable in low-risk category** (n = 1024)*^1^* | **p-value***^2^* |
| ICU stay (days) | 3.00 (0.00, 6.00) | 0.00 (0.00, 2.00) | **<0.001** |
| ICU days >1 | 21 (51%) | 263 (26%) | **<0.001** |
| ECMO | 0 (0%) | 14 (1.4%) | >0.9 |
| Ultrafiltration | 15 (37%) | 77 (7.5%) | **<0.001** |
| 30-day mortality | 3 (7.3%) | 14 (1.4%) | **0.025** |
| *^1^* Median (Q1, Q3); n (%) | | | |
| *^2^* Wilcoxon rank sum test; Pearson’s Chi-squared test; Fisher’s exact test | | | |

ECMO = extracorporeal membrane oxygenation; ICU = intensive care unit.

**Supplemental Table 13:** Effect of changes in risk category before surgery; time between the baseline NT-proBNP measurement and surgery more than 60 days.

| **Time of dynamic: >60d** | **Improvement** (n = 180)*^1^* | **Stable in high-risk category** (n = 142)*^1^* | **p-value***^2^* |
| --- | --- | --- | --- |
| ICU stay (days) | 1.0 (0.0, 5.0) | 3.0 (0.0, 7.0) | 0.093 |
| ICU days >1 | 80 (44%) | 79 (56%) | **0.046** |
| ECMO | 4 (2.2%) | 6 (4.2%) | 0.3 |
| Ultrafiltration | 33 (18%) | 41 (29%) | **0.026** |
| 30-day mortality | 3 (1.7%) | 6 (4.2%) | 0.2 |
| *^1^* Median (Q1, Q3); n (%) | | | |
| *^2^* Wilcoxon rank sum test; Pearson’s Chi-squared test; Fisher’s exact test | | | |

| **Time of dynamic: >60d** | **Deterioration** (n =  121)*^1^* | **Stable in low-risk category** (n = 1751)*^1^* | **p-value***^2^* |
| --- | --- | --- | --- |
| ICU stay (days) | 2.0 (0.0, 8.0) | 0.0 (0.0, 2.0) | **<0.001** |
| ICU days >1 | 65 (54%) | 442 (25%) | **<0.001** |
| ECMO | 11 (9.1%) | 37 (2.1%) | **<0.001** |
| Ultrafiltration | 38 (31%) | 158 (9.0%) | **<0.001** |
| 30-day mortality | 5 (4.1%) | 30 (1.7%) | 0.071 |
| *^1^* Median (Q1, Q3); n (%) | | | |
| *^2^* Wilcoxon rank sum test; Pearson’s Chi-squared test; Fisher’s exact test | | | |

ECMO = extracorporeal membrane oxygenation; ICU = intensive care unit.

**Supplemental Figure 1:** Flow chart

**
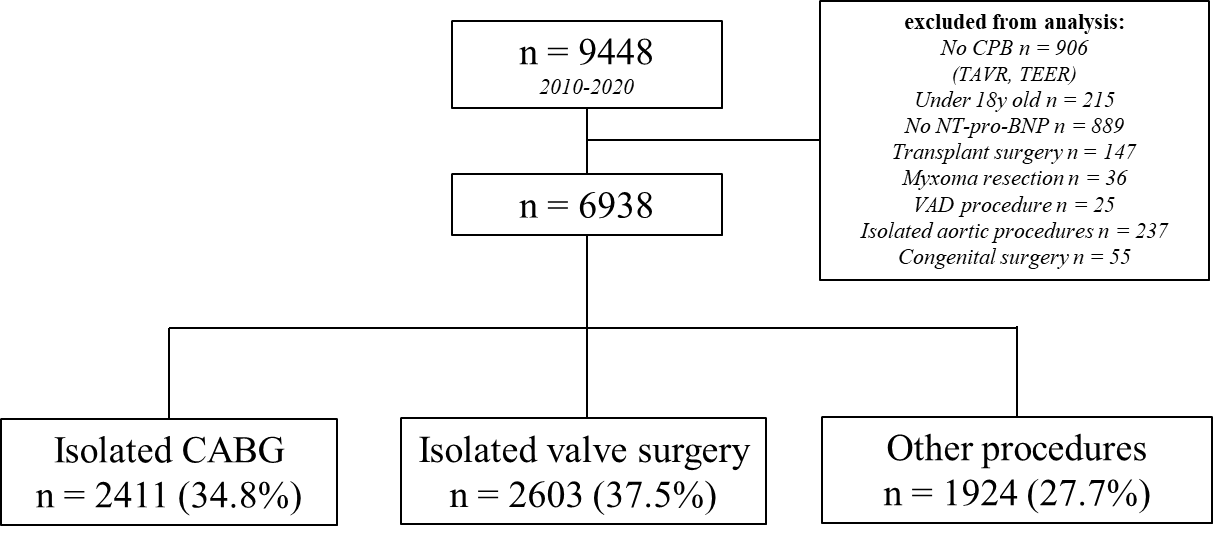
**

**Supplemental Figure 2:** ROC curves for preoperative NT-proBNP concentration and 30-day mortality

**
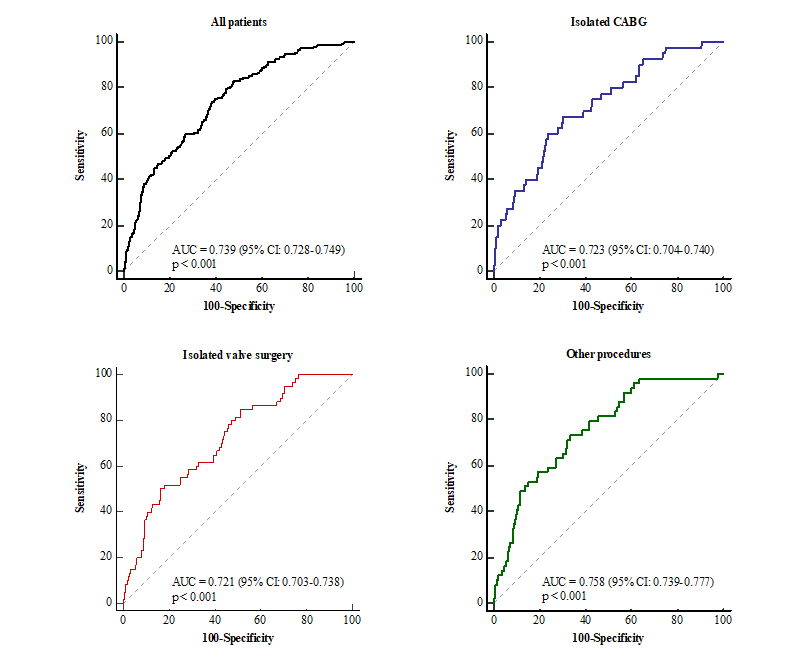
**

**Supplemental Figure 3:** Higher risk categories are associated with a worse postoperative outcome


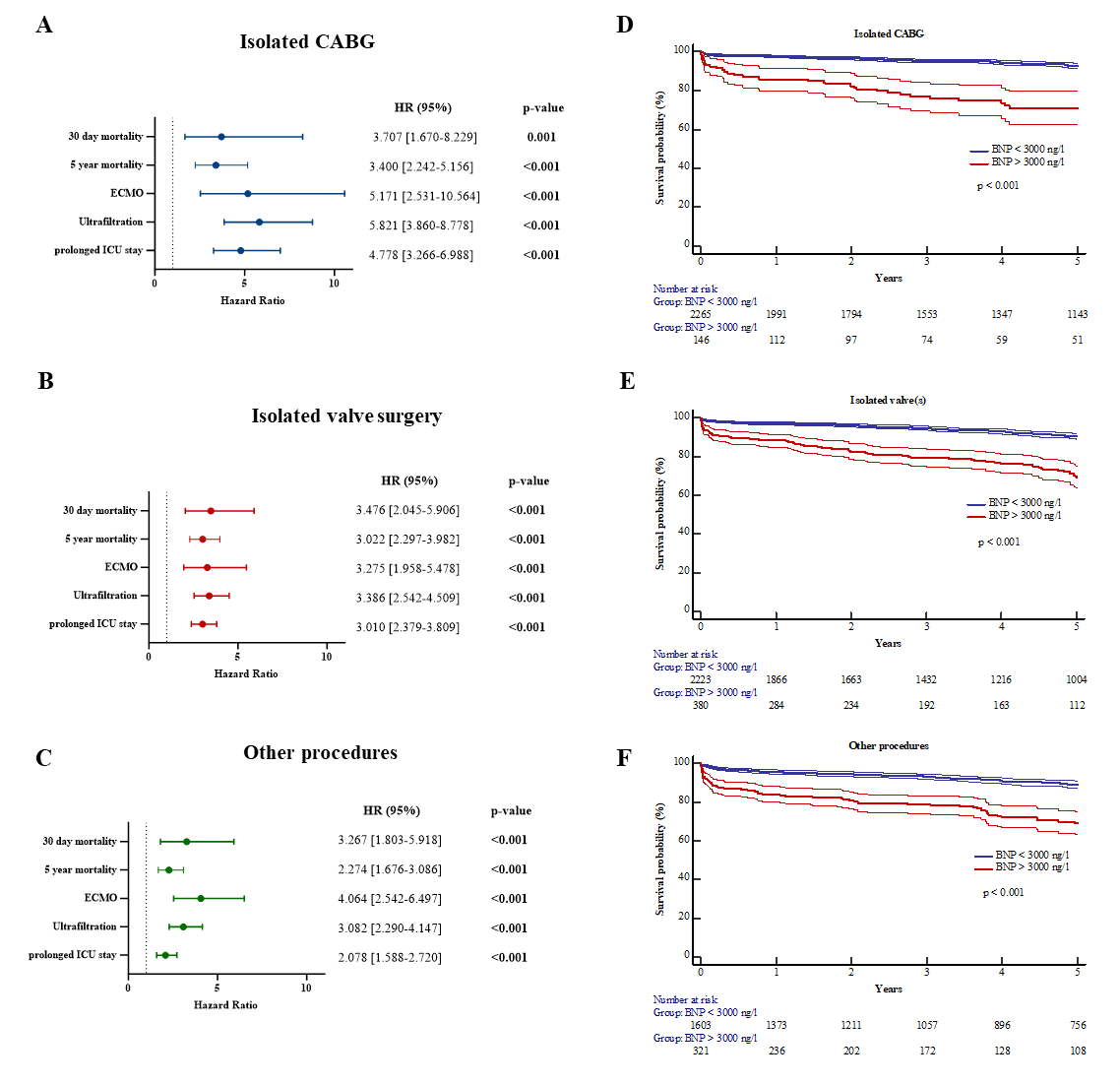


Patients in the high-risk category were compared with patients in the low-risk category. Regression models for **A** patients undergoing isolated CABG, **B** patients undergoing isolated valve surgery and **C** other patients were adjusted for EuroSCORE II. Kaplan-Meier curves for five-year mortality for **D** patients undergoing isolated CABG, **E** patients undergoing isolated valve surgery and **F** other patients. CABG = coronary artery bypass grafting; ECMO = extracorporeal membrane oxygenation; ICU = intensive care unit; prolonged ICU stay = longer than one day.
